# Prevalence, seroconversion and mother-to-child transmission of dual and triplex infections of HIV, hepatitis B and C viruses among pregnant women in Nigeria: study protocol

**DOI:** 10.1186/s12978-020-00995-8

**Published:** 2020-09-25

**Authors:** George Uchenna Eleje, Ikechukwu Innocent Mbachu, Uchenna Chukwunonso Ogwaluonye, Stephen Okoroafor Kalu, Chinyere Ukamaka Onubogu, Sussan Ifeyinwa Nweje, Chinwe Elizabeth Uzochukwu, Chike Henry Nwankwo, Preye Owen Fiebai, Olabisi Morebise Loto, Godwin Otuodichinma Akaba, Hadiza Abdullahi Usman, Ayyuba Rabiu, Richard Obinwanne Egeonu, Odion Emmanuel Igue, Bukola Abimbola Adesoji, Chiamaka Henrietta Jibuaku, Prince Ogbonnia Aja, Chiamaka Perpetua Chidozie, Hadiza Sani Ibrahim, Fatima Ele Aliyu, Aisha Ismaila Numan, Ogbonna Dennis Okoro, Solace Amechi Omoruyi, Ijeoma Chioma Oppah, Ubong Inyang Anyang, Aishat Ahmed, Shirley Nneka Chukwurah, Osita Samuel Umeononihu, Rebecca Chinyelu Chukwuanukwu, Eric Okechukwu Umeh, Ekene Agatha Emeka, Chukwuanugo Nkemakonam Ogbuagu, Ibrahim Adamu Yakasai, Oliver Chukwujekwu Ezechi, Joseph Ifeanyichukwu Ikechebelu, Triplex infection in pregnancy collaboration group

**Affiliations:** 1 Department of Obstetrics and Gynecology, Nnamdi Azikiwe University, Awka, Nigeria; 2 Department of Obstetrics and Gynecology, Nnamdi Azikiwe University Teaching Hospital, PMB 5025, Nnewi, Anambra State Nigeria; 3 Department of Pharmaceutical Sciences, Nnamdi Azikiwe University, Awka, Nigeria; 4 HIV Care Laboratory/HIV Care Department, Nnamdi Azikiwe University Teaching Hospital, Nnewi, Nigeria; 5 Department of Paediatrics, Nnamdi Azikiwe University, Awka, Nigeria; 6 Department of Nursing, Nnamdi Azikiwe University Teaching Hospital, Nnewi, Nigeria; 7 Department of Mass Communication, Nnamdi Azikiwe University, Awka, Nigeria; 8 Department of Statistics, Nnamdi Azikiwe University, Awka, Nigeria; 9 Department of Obstetrics and Gynecology, University of Port Harcourt, PortHarcourt, Nigeria; 10 Department of Obstetrics and Gynecology, University of Port Harcourt Teaching Hospital, PortHarcourt, Nigeria; 11 Department of Obstetrics and Gynecology, Obafemi Awolowo University, Ile Ife, Nigeria; 12 Department of Obstetrics and Gynecology, Obafemi Awolowo University Teaching Hospital Complex, Ile-Ife, Nigeria; 13 Department of Obstetrics and Gynecology, University of Abuja, Abuja, Nigeria; 14 Department of Obstetrics and Gynecology, University of Abuja Teaching Hospital, Abuja, Nigeria; 15 Department of Obstetrics and Gynecology, University of Maiduguri, Maiduguri, Nigeria; 16 Department of Obstetrics and Gynecology, University of Maiduguri Teaching Hospital, Maiduguri, Nigeria; 17 Department of Obstetrics and Gynecology, Bayero University, Kano, Nigeria; 18 Department of Obstetrics and Gynecology, Aminu Kano Teaching Hospital, Kano, Nigeria; 19 Department of Physiological Sciences, Obafemi Awolowo University, Ile-Ife, Nigeria; 20 Department of Nursing, Obafemi Awolowo University Teaching Hospital Complex, Ile-Ife, Nigeria; 21 Immunology Unit, Department of Medical Laboratory Science, Nnamdi Azikiwe University, Awka, Nigeria; 22 Department of Parasitology & Entomology, Faculty of Veterinary Medicine, University of Maiduguri Borno State, Maiduguri, Nigeria; 23 Department of Medicine, Faculty of Medicine, Nnamdi Azikiwe University, Awka, Nigeria; 24 Department of Radiology, Faculty of Medicine, Nnamdi Azikiwe University, Awka, Nigeria; 25 Department of Family Medicine, Faculty of Medicine, Nnamdi Azikiwe University, Awka, Nigeria; 26 Department of Medical Microbiology and Parasitology, Faculty of Medicine, Nnamdi Azikiwe University, Awka, Nigeria; 27 Nigerian Institute of Medical Research, Lagos, Nigeria

**Keywords:** Dual infection, HBV, HCV, HIV, Multiple infection, Nigeria, Seroconversion, Triplex infection

## Abstract

**Background:**

Nigeria contributes significantly to the global burden of HIV, Hepatitis B and C infections, either singly or in combinations, despite progress in HIV care regionally and globally. Although some limited data on mono infection of HIV, Hepatitis B and C virus infections do exists, that of dual and triplex infections, including seroconversion and mother-to-child transmission (MTCT) rates necessary for planning to address the scourge of infections in pregnancy are not available.

**Objectives:**

To determine the seroprevalence, rate of new infections, MTCT of dual and triple infections of HIV, Hepatitis B and C viruses and associated factors, among pregnant women in Nigeria.

**Method:**

A multicenter prospective cohort study will be conducted in six tertiary health facilities randomly selected from the six geopolitical zones of Nigeria. All eligible pregnant women are to be tested at enrollment after informed consent for HIV, Hepatitis B and C virus infections. While those positive for at least two of the infections in any combination will be enrolled into the study and followed up to 6 weeks post-delivery, those negative for the three infections or positive for only one of the infections at enrolment will be retested at delivery using a rapid diagnostic test. On enrolment into the study relevant information, will be obtained, and laboratory test of CD4 count, liver function test and full blood counts, and prenatal ultrasonography will also be obtained/performed. Management of mother-newborns pairs will be according to appropriate national guidelines. All exposed newborns will be tested for HIV, HBV or HCV infection at birth and 6 weeks using PCR technique. The study data will be documented on the study case record forms. Data will be managed with SPSS for windows version 23. Ethical approval was obtained from National Health Research Ethics Committee (NHREC) (NHREC/01/01/2007–23/01/2020**)**.

**Conclusion:**

Pregnant women with multiple of HIV, HBV and HCV infections are at increased risk of hepatotoxicity, maternal and perinatal morbidity and mortality. Additionally, infected pregnant women transmit the virus to their unborn baby even when asymptomatic. Children born with any of the infection have significantly poorer quality of life and lower five-year survival rate. Unfortunately, the seroconversion and MTCT rates of dual or triplex infections among pregnant women in Nigeria have not been studied making planning for prevention and subsequent elimination of the viruses difficult. The study is expected to fill this knowledge gaps. Nigeria joining the rest of the world to eliminate the triple infection among children rest on the availability of adequate and reliable data generated from appropriately designed, and powered study using representative population sample. The establishment of the three-in-one study of prevalence, rate of new infection, rate and risk factor for MTCT of dual and triple infection of HIV, Hepatitis B and C viruses among pregnant women in Nigeria is urgently needed for policy development and planning for the improvement of the quality of life of mothers and the elimination of childhood triplex infection.

## Plain English summary

Nigeria has large share in the number of persons suffering from HIV, Hepatitis B and C virus infections, either as one infection or combination of the infections. The details of the infections when they are seen in combinations during pregnancy including new acquired infections during pregnancy and transfer of the infections from mother to the baby are not available.

The aim of the study is to find out the number of pregnant women carrying the combined infections of HIV, Hepatitis B and C viruses, new infection rates and rate of transfer of the infection from mother to baby in Nigeria.

A multicenter study will be conducted in six tertiary hospitals selected randomly in all the zones of Nigeria. All qualified pregnant women are to be tested at enrollment after informed consent for HIV, Hepatitis B and C virus infections. While those positive for at least two of the infections in any combination will be allowed to participate in the study and followed up to 6 weeks after delivery, those negative for the three infections or positive for only one of the infections at inclusion in the study will be retested at delivery using a rapid diagnostic test. On enrolment into the study, relevant information will be obtained, and laboratory test of CD4 count, liver function test and full blood counts, and ultrasonography will also be done. Management of mother-newborns pairs will be according to appropriate Nigeria guidelines. All newborns whose mothers test positive to any of the three infections will be tested for HIV, HBV or HCV infection at birth and 6 weeks of life. The study data will be documented on the study forms and later analysed. Ethical approval was obtained from National Health Research Ethics Committee.

Pregnant women with multiple of HIV, HBV and HCV infections are at increased risk of hepatotoxicity, maternal and perinatal morbidity and mortality. Additionally, infected pregnant women can transmit the virus to their unborn baby even when asymptomatic. Children born with any of the infections have significantly poorer quality of life and lower five-year survival rate. Unfortunately, studies on turning from negative tests to positive tests for the infections during pregnancy and transfer of the infection from mother to baby of any of the combined infections among pregnant women in Nigeria have not been conducted making planning for prevention and subsequent elimination of the viruses difficult. The study is expected to fill this knowledge gaps. Nigeria joining the rest of the world to eliminate the triple infection among children rest on the availability of adequate and reliable data generated from appropriately designed, and powered study using representative population sample. The establishment of the prevalence, rate of new infection, rate and risk factors for transfer of the infection from mother to baby for combined HIV, Hepatitis B and C viruses among pregnant women in Nigeria is urgently needed for policy makers and planning for the improvement of the quality of life of mothers and the elimination of childhood combined infection.

## Background

The dual (any two of Human Immune Deficiency Virus (HIV), Hepatitis B virus (HBV), and Hepatitis C virus (HCV)) or triplex (combined HIV, HBV, and HCV) infections occur worldwide with the highest prevalence in sub Saharan Africa. In 2019, United State Agency for International Development (USAID) and the Nigeria National Agency for the Control of AIDS (NACA) estimated that there were 1.9 million people living with HIV in Nigeria, giving a National HIV prevalence of 1.4% among adults of reproductive age group [1].

Similarly, more than 2 billion people worldwide are estimated to have had hepatitis B virus (HBV) infection, with 350–400 million being chronic carriers of the virus [2]. Although, the prevalence varies throughout the world, it is highest in tropical region of the world [3]. It is estimated that 5–15% of adults in Sub-Saharan Africa are chronically infected with HBV. A national survey in Nigeria in 2016 reported prevalence rate of 12.2% [4]. HCV on the other hand affects about 3% of the world’s population; that is, 170 million people globally [5]. In Nigeria, the estimated prevalence of HCV infection varies widely (0.4–14.7%) depending on the region and subpopulation being considered [6, 7]. A common denominator among all the reported prevalence in Nigeria is the lack of generalizability as their data is not nationally representative.

Dual or triplex infection of these viruses in a pregnant woman is a common occurrence because of the shared modes of transmission such as blood transfusion, sharing of sharp objects, and unsafe sex [8]. Thus this poses significant challenge to the unborn child because of the propensity to be transmitted vertically. Secondarily, it is an occupational hazard to service providers. When the three infections coexist, which is common, there is an increased risk of maternal complications, vertical and horizontal transmission to newborn, partners and health service providers respectively [9]. In addition, the progression from HIV to AIDS is faster in people triply infected with HBV and HCV, resulting in the swift worsening of immune system that accompanies HBV and HCV infections [10].

In sub-Saharan Africa, HIV, HBV, and HCV infections are endemic especially among the reproductive age group with high fertility rate, making vertical transmission a major cause for concern [11]. Unlike HIV, HBV and HCV testing is not routinely done during pregnancy in Nigeria and thus most pregnant women with HBV and HCV are undiagnosed and unknown [12]. Vertical transmission of HBV and HCV is thus the norm rather than the exception, as no intervention is provided for these women. In addition, the unknown when coinfected with HIV are treated with antiretroviral Lamivudine monotherapy leading to the development of drug resistance with the attendant morbidity, mortality and increased rate of mother-to-child transmission (MTCT) [13]. Despite progress in HIV care globally and regionally, Nigeria contributes significantly to the global burden of these triplex infections. A major cause for concern is that what is known about these infections especially HBV and HCV are majorly based on estimates and extrapolations, making national planning and decision to eliminate these infections almost impossible.

The World Health Organisation (WHO) recommends screening of all HIV patients for viral hepatitis, vaccination against HBV in non-immune individuals and providing anti HBV therapy in HIV/HBV dually or triply infected patients [14]. Adoption of this strategy will reduce MTCT of these viruses to the children of infected mothers. Despite the marginal progress made in the control of HIV, 32% of the global gap in MTCT is from Nigeria, the contribution made by HBV and HCV where little or nothing have been done is not known and could only be imagined [15].

Children who are perinatally bared to these viruses are at increased risk of early morbidities, which in turn are associated with fetal and neonatal hepatitis, chronic HBV infection, liver cirrhosis and primary liver cell carcinoma [16, 17]. A quarter of children infected with HBV often die from HBV related chronic liver diseases in adulthood [18]. Correspondingly, chronic HCV infection is associated with increased incidence of preterm delivery, intrauterine growth restriction and vertical transmission [19]. About 20% of children vertically infected with HCV develop active infection [20]. In addition, following childbirth, most untreated HIV infected children do not live to see their first birthday, with greater rapid deterioration in those coinfected with Hepatitis B and C viruses [21–23].

Elimination of MTCT of HIV and hepatitis B and C virus infections in Nigeria requires the implementation of feasible, culturally acceptable and sustainable interventions and policies that is based on adequate and reliable in-country evidence and data. Unfortunately, a nationally representative data on prevalence of triplex infection, rate of new infection and MTCT rate based on appropriately design and powered study is lacking. This study is designed and powered to bridge this knowledge gap and facilitate national planning and policy development towards the elimination of childhood triplex infection. In addition, this study is in line with Sustainable Development Goal number 3 for the improvement of maternal and child health, as well as the WHO strategy on the determination of the prevalence of dual and triplex infection of HIV, HBV and HCV infections which will serve as the baseline data [11].

## Objectives

This study is designed to establish the baseline data for the prevalence, rate of new infection, rate and risk factor for mother-to-child transmission of dual and triple infection of HIV, Hepatitis B and C viruses among pregnant women in Nigeria necessary for policy development and planning for the improvement of the quality of life of mothers and the elimination of childhood triplex infection.

### Specific objectives

1. To determine the seroprevalence of the dual and triplex infection among pregnant women in Nigeria.
2. To assess the hepatic enzyme status, and patterns among pregnant women with dual/triplex infections.
3. To determine the new infection rate (seroconversion) and risk factors for seroconversion of dual and triplex infections among pregnant women in Nigeria.
4. To determine the rate of, and sociobiological risk factor for mother-to-child transmission of dual/triplex infections using PCR at 6 weeks post-delivery.

## Methodology

### Study design

**Fig. 1:**
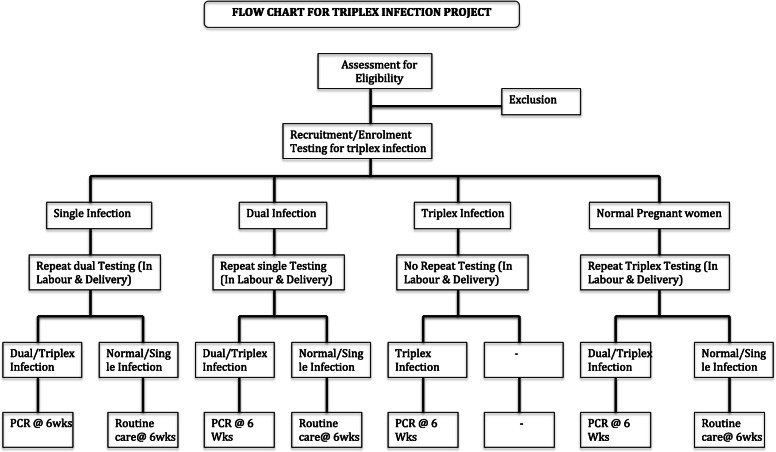
Flow-chart

The study will utilize a multicenter prospective cohort design to determine the seroprevalence, new infection rate, the rate and risk factors for mother- to- child transmission of the triplex infection in pregnancy using polymerase chain reaction (PCR) at birth and 6 weeks post-delivery in Nigeria (Fig. 1).

### Study population

The study will be conducted among mother-infant pairs drawn from pregnant women attending antenatal care in randomly selected tertiary centre in each of the six geopolitical zones of Nigeria.

### Study sites

Participants will be recruited from one randomly selected tertiary level facility in each of the five geopolitical zones in Nigeria apart from the south east zone where the lead institution for the TETFund National Research Fund 2019 is situated. The sites randomly selected in addition to the lead Institution Nnamdi Azikiwe University Teaching Hospital, Nnewi [South-East] are;

1. University of Maiduguri Teaching Hospital, Maiduguri [North West].
2. Aminu Kano Teaching Hospital, Kano [North East]
3. Obafemi Awolowo University Teaching Hospital Complex, Ile-Ife [South-West]
4. University of Port Harcourt Teaching Hospital, Port Harcourt [South-South]
5. University of Abuja Teaching Hospital, Gwagwalada [North-Central]

### Eligibility criteria

#### Inclusion criteria

All pregnant women receiving antenatal care services at the six randomly selected tertiary hospitals in Nigeria as well as their newborn infants, who did not have any of the exclusion criteria.

### Exclusion criteria

#### Mothers

Women whose pregnancy could not be confirmed by ultrasound or blood pregnancy test.

#### Newborns

Infants identified to have gross congenital malformation at birth.

### Sample size determination

The sample size was determined based on the Cochran’s formula for sample size determination when the population is more than 10,000. The sample size was obtained using the Cochran’s formula [24] N = Z^2^alphaPQ/d^2^, where: Z = standard normal deviation at 95% confidence interval; P = prevalence of the problem (prevalence of health facility delivery rate among pregnant women in Nigeria based on a recent study by Adedokun and Uthman that utilized data from 2013 Nigeria Demographic and Health Survey involving 20,192 women who had delivery within 5 years of the survey was put at 38.0% [25]); Q = 1-p and d = 0.05. The ultimate was adjusted to allow a non-inferiority sample size of 363 obtained and rounded up to 436 to cater for 20% attrition or non-response. Expectedly, at least four hundred and thirty six pregnant women will be recruited from each selected facility.

#### Sampling technique

One tertiary hospital was selected from each of the five geopolitical zones in Nigeria by simple random sampling using lottery method apart from the lead institution. In each selected hospital, all consecutive eligible pregnant women who give informed consent will be recruited until sample size is attained.

### Study procedure

#### Pregnant mother

After consenting process, eligible mothers will be privately interviewed at enrollment (during the booking) and at delivery to obtain relevant socio-demographic characteristics, obstetric and medical history. The information will also include address, age, marital status, religion, highest educational attainment, occupation, parity, and gestational age, number of ANC visits, hepatitis B vaccination history, antenatal sexual activity history, anthropometry, as well as HIV, HBV and HCV status, sexual history of the partner, and use of anti-retroviral drugs. All study related information will be entered into Case Record Form (CRF) designed for the study. Gestational age (GA) at enrolment and expected date of delivery will be based on mother’s last menstrual period (LMP) or first trimester ultrasound for women with unsure LMP.

The enrollees will be screened for HIV, HBV, and HCV at enrolment during the booking for ANC using a rapid diagnostic test. Repeat screening will be performed at term or on admission in labor / scheduled delivery for those who screen negative to at least two infections at enrolment. All mothers with at least two infections at enrolment will receive prenatal ultrasonography at 28 to 32 weeks GA to determine fetal wellbeing. Mothers who test positive will be managed according to the national guidelines for the treatment of their infection and prevention of mother-to-child transmission of the infection.

In addition to the routine antenatal follow-up visits for all pregnant women, the participants in the study will in addition be seen at 28–32 weeks and 6 weeks’ post-partum. Participants will be reminded of their appointments 2 to 3 days prior to the scheduled visits through a phone call so as to forestall lost to follow-up. The Home-Based Care team will be notified as soon as possible of any defaulting client for tracking. Delivery method will be based on obstetric indication and according to prevailing national guidelines.

#### Newborn infants

The infants of participating mothers will be enrolled into the infant arm of the study on delivery and follow-up for 6 weeks. All the infants will benefit from appropriate prophylaxis, vaccination, infant feeding counseling and support, and follow-up. Infants sex, anthropometry (weight, length, occipitofrontal circumference (OFC)) at delivery and follow-up, type of post-exposure prophylaxis received and actual infant feeding practice. The obtained information will also be entered into the CRF. Depending on their exposed status, blood sample will be collected from the infants at birth and 6 weeks for HIV, HCV and HBV status determination using polymerase chain reaction technique. Results will be communicated to the parents after adequate counseling and the affected newborn will be linked for appropriate care and treatment. Neonatal antiretroviral prophylaxis and management will be instituted for all exposed neonates in conjunction with a neonatologist/infectious disease pediatrician according to national guidelines.

### Laboratory procedures

#### Maternal HIV, hepatitis B and C virus screening and testing

Ten millimeters of venous blood will be collected in EDTA bottle labeled with participants identifiers by trained phlebotomist, and will be used to carry out the three rapid ELISA tests. Participants who tested negative at baseline will be rescreened for HIV, Hepatitis B and C viruses at delivery or labour. Serial rapid HIV testing will be done according to Nigerian National HIV testing guidelines viz. *Alere Determine HIV*-1/2 (*Alere* Medical Co. Ltd., Matsudo, *Japan*) test kit as a screening test, followed by the Uni-Gold Recombigen® *HIV*-1/2 (Trinity Biotech, Ireland) assay if positive and finally confirmed by HIV1/2 STAT-PAK (Chembio Diagnostic Systems, Inc., USA). The HBsAg and anti-HCV will be tested using ELISA kit manufactured by LabACON (Hangzhou Biotest Biotech Company, Ltd., China) which has specificity of 99.0% and a sensitivity of 99.9% according to company declared figures. The kit has in-built controls. The manufacturer’s instruction will be strictly followed. The results will be reported as positive or negative.

#### HIV, hepatitis B and C viral load determination

HIV, HBV and HCV viral load will be estimated using ROCHE COBAS CTM/CAP REAL-TIME PCR. All blood specimen for Viral load test and infant status determination will be stored in the minus 25-degree freezer after separation at the sites. The stored samples will be batched transported under cold chain condition for analysis at the Molecular Virology Laboratory at Nnamdi Azikiwe University Teaching Hospital (NAUTH), Nnewi, Anambra State, Nigeria. Fifteen militres of blood will be collected into an EDTA anticoagulant vacutainer tube and gently mixed by inversion. The blood will be centrifuged at 1800 rpm for 25 min. The plasma will be transferred into a 2 ml cryovial tube and stored at − 25 degree centigrade until when enough samples are pooled for testing. Before analysis, frozen specimens will be brought to room temperature until it is completely thawed and vortexed for 10–15 min. The controls will be removed and allowed to equilibrate before use. A vortex mixer will be powered on and set at 56 °C. The 1100 μl of the plasma sample will be dispensed into SPU tubes and placed unto the vortex mixer for 10–15 min. The negative, low positive and high positive controls will be pipetted into SPU tubes accordingly.

##### Workflow

The COBAS TaqMan Analyzer 48 or 96 Analyzer will be started within 120 min following completion of specimen and control preparation. The COBAS TaqMan 48 analyzer automatically determines the HBV DNA concentration for the specimens and controls. The DNA concentration will be expressed in copies/mL. The Cycle Threshold value (Ct) for the HBV DNA and the HIV QS DNA will be determined. The HBV DNA concentration based upon the Ct values for the HBV DNA and the HIV-IQS DNA and the lot-specific calibration coefficients provided on the cassette barcodes would also be determined.

##### Results validation

After the COBAS TaqMan 48 analyzer run, the results will be checked for flags or error messages in the result report. Specimens with flags and comments would be interpreted as described in the results section. After accepting, the results would be stored in the data archive. All the used K-tubes would be removed from the COBAS TaqMan 48 Analyzer.

The results would be validated or invalidated depending on whether there is an appearance of a flag or not on any of the controls [HBV L (+) C, v2.0, and CTM (−) C].

### Infant HIV, HBV and HCV diagnosis

Determination of the HIV status of the exposed infant will be done in accordance with current Nigeria National Guideline for PMTCT. All HIV, HBV, HCV exposed infants will be tested using DNA PCR by collecting the dried blood sample (DBS) at birth and age of 6 weeks. The dried blood sample will be analysis centrally using PCR at Molecular Virology Laboratory, NAUTH, Nnewi, Anambra State, Nigeria.

### Ethics approval

Approval was obtained from the National Health Research Ethics Committee (**NHREC/01/01/2007–23/01/2020).** The recruiting hospitals also obtained concurrent approvals from their local ethics committees before commencement of the study according to the National Ethics Code. Written informed consent will be obtained from all eligible pregnant women before enrollment into the study.

### Pre-testing of study data collection tools

All the data collection tools will be jointly developed by the study investigators, and sent to obstetrician, pediatrician, biostatistician and infectious diseases expert, and revised to ensure internal validity. Study questionnaire will be pretested through administration to 20 pregnant women in a hospital center other than the center included in the sample. Necessary corrections and adjustments will be carried out based on the feedback received. Study data will be collected by trained research assistants under the supervision of the investigators.

### Data management plan

The study’s CRFs will be used to collect the study specific information by the research assistants. Information on socio-demographic characteristics, obstetrics and medical history, clinical examination findings, antenatal follow up and laboratory investigation results will be collected. The information on the CRF will be doubly-entered into a computer using, cleaned and analysed with Statistical Package for Social Sciences (SPSS) version 23. Univariate analysis will first be conducted to determine the prevalence, rates of new infection, rates and MTCT for HIV, HBV and HCV, followed by bivariate and multiple logistic regression to determine the risk factors for MTCT and new infections. Appropriate statistical test will be used as appropriate. The result outputs will be displayed as tables and figures.

## Discussion

The study is designed to determine the seroprevalence, new infection rate, the rate of and risk factors for mother-to-child transmission (MTCT) of the dual and triplex infection in pregnancy using PCR at 6 weeks post-delivery in Nigeria.

Pregnant women with dual or triplex of HIV, HBV and HCV infections are at increased risk of hepatotoxicity, with attendant higher risk of maternal and perinatal morbidity and mortality. Infected pregnant women can transmit the virus to their unborn baby even when asymptomatic. Children born with any of the infections have significantly poorer quality of life and lower five-year survival rate. Unfortunately, the incidence, and MTCT rates of dual or triplex infections among pregnant women in Nigeria is not known making planning for prevention and subsequent elimination of the infections difficult. This study is expected to fill the knowledge gaps.

Nigeria joining the comity of the nations to eliminate the triple infection among children rest on the availability of adequate and reliable data generated from appropriately designed, and powered study using representative population sample. The establishment of the prevalence, rate of new infection, rate of and risk factor for MTCT of dual and triple infection of HIV, Hepatitis B and C viruses among pregnant women in Nigeria is urgently needed for policy development and planning for the improvement of the quality of life of mothers and the elimination of childhood triplex infection.

The study will be conducted in the six geopolitical zones with their distinct cultural, religious and behavior characteristics thus will provide adequate data for policy and planning for the country. However, a possible weakness of the study is that it will be conducted in tertiary institutions, which may make generalization difficult. However, the current weakness in the Nigerian health system have made it possible for women to present first at the Teaching hospital without passing through primary and secondary health facilities. The sociobiological and obstetrics characteristics of pregnant women seen at the teaching hospital in Nigeria are similar to those seen at the secondary and primary facilities [26]. This makes generalization of the results possible for the general population.

A major strength of this study is that is one of the few studies in the country that will be measuring the incidence of HIV, Hepatitis B and C viruses in pregnancy using data from all the six zones of the country. In addition, approval was obtained from the national Ethics community domicile at the Department of Health Planning, Research and Statistics, Federal Ministry of Health, which will make translation to policy easy. Furthermore, almost of all the investigators are members of the Society of Gynecology and Obstetrics of Nigeria (SOGON) will also make translation to Practice Guideline easy.

## Data Availability

No data were generated during the current status of the study. Once the study is finalized and the results are published, a specific procedure for obtaining access to the database will be made.

## Abbreviations

AIDS: Acquired immunodeficiency syndrome
CRF: Case Record Form
DBS: Dried blood Sample
DNA: Deoxyribonucleic acid
EDTA: Ethylene diamine tetraacetic acid
ELISA: Enzyme Linked Imunosorbent Assay
GA: Gestational age
HBV: Hepatitis B virus
HCV: Hepatitis C virus
HIV: Human Immuno Deficiency Virus
LMP: Last menstrual period
MTCT: Mother-to-child-transmission
NACA: National Agency for the Control of AIDS
NAUTH: Nnamdi Azikiwe University Teaching Hospital
NHREC: National Health Research Ethics Committee
PCR: Polymerase Chain Reaction
PMTCT: Prevention of Mother-to-Child-Transmission
SDG: Sustainable Development Goal
SPSS: Statistical packages of social sciences
USAID: United State Agency for International Development
WHO: World Health Organization
